# Inflammatory Markers and Plasma Lipids in HIV Patients: A Correlation Analysis Study

**DOI:** 10.2174/1874091X01711010105

**Published:** 2017-12-28

**Authors:** Rudo Muswe, Olav Oktedalen, Danai T. Zhou, Enita Zinyando, Sandra Shawarira-Bote, Babill Stray-Pedersen, Atipa Siziba, Zvenyika A.R. Gomo

**Affiliations:** 1 Department of Chemical Pathology, University of Zimbabwe, Harare, Zimbabwe; 2 Department of Infectious Diseases, Oslo University Hospital, Oslo, Norway; 3 Department of Medical Laboratory Sciences, University of Zimbabwe, Harare, Zimbabwe; 4 Newlands - Clinic Harare, Harare, Zimbabwe; 5 University of Oslo, Division of Women and Children, Oslo, Norway; 6 Controlnet Consulting - Consultants Midrand, Gauteng, South Africa

**Keywords:** ART, CHD, Cytokines, HIV, HsCRP, IL-10, Inflammation, Lipids, TNF-alpha

## Abstract

**Background:**

Recent evidence suggests that HIV infection, even with treatment, increases the risk of coronary heart disease (CHD) and that both chronic inflammation and traditional risk factors play key roles in HIV-associated CHD.

**Subjects and Methods:**

Patients (N=152), attending Harare HIV clinic, 26% of them male and 82% of them on antiretroviral therapy (ART), were studied. Inflammatory markers comprising of cytokines such as pro-inflammatory tumor necrosis factor-α, (TNF-α), anti-inflammatory interleukin 10, (IL-10) and highly sensitive C reactive protein (hsCRP) together with lipids were assayed using enzyme linked immunosorbent assay (ELISA), immuno-turbidimetric and enzymatic assays, respectively. Correlation analysis of inflammatory markers versus lipid profiles was carried out using bivariate regression analysis.

**Results:**

Anti-inflammatory cytokine IL-10 and inflammatory hsCRP levels were elevated when measured in all the HIV positive patients, while TNF-α and lipid levels were within normal ranges. Pro-inflammatory TNF-α was significantly higher in ART-naive patients than ART-experienced patients, whereas the reverse was observed for anti-inflammatory IL-10 and anti-atherogenic HDL-C. Correlation analysis indicated a significant positive linear association between IL-10 and total cholesterol (TC) levels but no other correlations were found.

**Conclusion:**

High cytokine ratio (TNF-α/IL-10) indicates higher CHD risk in ART-naive patients compared to the ART-exposed. The CHD risk could be further strengthened by interplay between inflammatory markers and high prevalence of low HDL-C. Lack of correlation between pro-inflammatory markers (hsCRP and TNF-α) with lipid fractions and correlation between anti-inflammatory IL-10 with artherogenic TC were unexpected findings, necessitating further studies in future.

## 1 BACKGROUND

Sub-Saharan Africa remains the continent most affected by HIV and fortunately antiretroviral therapy (ART) has also become more accessible to HIV patients in this part of the world [1]. There is, however, growing concern of metabolic complications associated with HIV and its treatment, which may increase coronary heart disease (CHD) risk [2] as patients live longer with their disease and increase their likelihood of developing long term health complications including CHD [2]. Moreover, HIV infection is an inflammatory disease that results in chronic immune activation and inflammation [3]. This together with the characteristic dyslipidemia associated with HIV and its treatment may contribute to the increased CHD risk observed in HIV positive patients [4]. Emerging studies report increased CHD risk among HIV infected populations in sub Saharan Africa. Gomo *et al*. reported modest increased CHD risk in Zimbabweans when switching from first line to second line drugs [5] whereas Muhammad *et al.*, reported high prevalence of hypertension, obesity and metabolic syndrome in Nigerian patients associated with ART exposure [6, 7]. A recent Zimbabwean study also reported an increasing number of patients with traditional risk markers of CHD such as high atherogenic lipids and hypertension [8].

Inflammation is implicated in almost every health problem and its consequences tend to be worse for people with HIV since the ongoing presence of the virus maintains CD4 and CD8 T-cells in a constant state of activation [9]. Several factors likely contribute to persistent inflammation; these include residual HIV replication, persistent virus expression, loss of immunoregulatory cells, collagen deposition, microbial translocation, chronic co-infections, and thymic dysfunction [10]. How these factors influence disease outcomes and how chronic inflammation should be managed during therapy are the focus of intense ongoing investigation [10].

HIV proteins, including TAT, NEF, VPR, and gp120, directly stimulate the immune response by altering cytokine signaling [11]. Additionally, the lumen of the gastrointestinal (GI) tract contains an enormous quantity of bacterial species that establish symbiotic relationships with human host immune system and epithelial cells [12]. This reduces risk of microbial translocation (MT) from the lumen of GI tract to the systemic circulation in the general population. However, various studies have suggested a role for MT in HIV pathogenesis and have proposed that MT contributes to systemic immune activation in people with HIV, which helps explain the pathogenesis of non-AIDS-related morbidity, including cardiovascular disease [12].

Furthermore, protease inhibitors are associated with elevated levels of total cholesterol (TC), low density lipoprotein cholesterol (LDL-C) and triglycerides, as well as insulin resistance and abdominal fat accumulation, factors that promote inflammation and hence atherosclerosis [13]. Other groups of ART drug regimens have similar atherogenic effects, albeit to varying degrees [8]. Long-term ART exposure has been associated with increased risk of dyslipidemia and CHD, particularly for combinations that are protease inhibitor-based [14]. In addition, ART may indirectly play a role in inflammation through its effects on metabolism though it is not clear, if antiretroviral drugs directly cause inflammation [15].

Atherosclerosis is a chronic inflammatory disease of the arterial wall [16] and inflammation is a crucial link between HIV and CHD. When the CD4 lymphocytes are infected by HIV, a systemic inflammatory response is triggered [17]. Pro-inflammatory cytokines such as TNF-α and IL-6 are released systemically and locally at the coronary arteries. The liver is in turn stimulated to produce acute phase proteins such as C-reactive protein, plasminogen and fibrinogen [18].

 This results in endothelial dysfunction thus promoting formation of atherosclerotic plaques and CHD [17]. Elevated levels of pro-inflammatory cytokines have been associated with cardiovascular mortality in patients with HIV [19]. In particular, TNF-α has been implicated in CHD ranging from myocardial dysfunction to recurrent coronary events after myocardial infarction [19].

In response to inflammation, the body produces anti-inflammatory cytokines such as IL-10 and IL-4 in an attempt to counteract the inflammation [18]. Past studies showed a significant increase in TNF-α/IL-10 ratio in patients with CHD compared to control subjects [19]. Additionally, it is well-established that metabolic changes associated with ART may promote atherosclerotic plaque formation [17]. However, more clinical and experimental data are needed to establish whether ART can reduce inflammation. The association between HIV infection itself and the adverse effects of ART with respect to CHD is still not clear [20].

In our study, we expect a high CHD risk when evaluated by inflammatory markers (high TNF-α/IL-10 ratio, high hsCRP) and deranged serum lipid levels in HIV-infected patients; independent of ART experience. We determined the levels of inflammatory markers and lipid levels in HIV-infected adults attending a treatment clinic in Harare, Zimbabwe, and assessed correlations between the levels of systemic inflammation (hsCRP, TNF-α and IL-10) and lipid levels in the context of CHD risk in both ART positive and ART negative patients.

## 2 MATERIALS AND METHODS

### 2.1 Study Design, Ethical Approval and Study Setting

The study participants were HIV positive male and female adults above 18 years of age, recruited from an HIV Outpatients Clinic in Harare, Zimbabwe, who were attending the clinic for routine check-up and antiretroviral (ARV) therapy. ARV therapy included first line drugs which contained two nucleoside reverse transcriptase inhibitors (NRTIs) and a non-nucleoside reverse transcriptase inhibitor (NNRTI) as well as second line therapy which involved a switch from the first line NNRTI to a protease inhibitor (PI) and alternated NRTIs. The study participants comprised of three groups: naïve group (n=27), first line group (n=114) and second line group (n=11). The first line group had three subgroups of different drug combinations: tenofovir (TDF) + nevirapine (NVP) + lamivudine (3TC) (n=91); TDF + efavirenz (EFV) + 3TC (n=17) and stavudine/zidovudine (STV/ZDV) + NVP + 3TC (n=6). The second line group had individuals taking the following drug combinations: TDF+ atazanavir (ATV) + ritonavir (RTV) + one nucleoside analogue (one more NRTI) or abacavir (ABC) + ATV + 3TC or TDF + ATV + 3TC [8, 21]. The study was ethically cleared by the Joint Research Ethics Committee of the University of Zimbabwe, College of Health Sciences and the Parirenyatwa Group of Hospitals (JREC), the Medical Research Council of Zimbabwe (MRCZ) and the Research Ethics Committee, (REK), Norway. Initial recruitment, study setting, sample and data collection are described fully elsewhere [8, 21].

 Patients on lipid lowering drugs and anti-inflammatory drugs, or those with documented, current tuberculosis, hepatitis B, hepatitis C, meningitis, arthritis and prior history of documented CHD were excluded. The above patients were excluded because they could be having acute inflammation associated with their co-morbidities. On the one hand, this study sought to measure chronic, sub-clinical inflammatory markers, known to potentially contribute to coronary heart disease. On the other hand, the restriction of participants to a narrow subset reduced potential confounding from opportunistic infections, although it lowered generalisability of the results to HIV-infected adults with the various co-morbidities.

### 2.2 Sample Size Calculation

Sample size was calculated using Dobson’s formula:

N= Z^2^ p (1-p)/e^2^ where Z=standard normal deviate corresponding to two sided significant level. i.e. 1.96 at 95% CI, e = margin of error =5%, p = prevalence of CHD risk in HIV patients = 11.1% [22]. Hence, N= minimum sample size = 152

### 2.3 Laboratory Assays

#### 2.3.1 Measurement of Cytokines

Pro-inflammatory TNF α and anti-inflammatory IL-10 levels were measured on 152 samples that were randomly selected from samples collected at the nine months’ visit using commercial ELISA kits (WKEA Med Supplies Corporation, Changchun, China) and an ELISA ELX 800 universal microplate reader (Biotech Instruments, Highlands Park, Winooski, USA). Controls with known concentration were used to validate the assays and the measured coefficient of inter-assay variation was less than 10% for both manual ELISA methods. A within run method validation was done and coefficients of intra-assay variation for the TNF-α and IL-10 ELISA manual methods were 5.8% and 6.4% respectively [23]. The limit of detection was 0·5 pg/ml k(IL-10) and 3 pg/ml (TNF-α).

### 2.4 Measurement of HsCRP

Highly sensitive C-reactive protein (hsCRP) was measured on 65 randomly selected samples collected at nine months’ follow-up due to shortage of resources for this assay. Samples were analysed at Harare Hospital Clinical Chemistry Laboratory using Siemens Dimension Xpand automated analyser (Siemens Healthcare Diagnostics Inc, Newark, DE, USA). The Diazyme C-reactive protein extended method is based on a latex-enhanced immunoturbidimetric assay, which has a linear range of 0.20 - 20 mg/L that extends below the measurement range typical of most conventional CRP assays. This lower range of measurement includes the evaluation of conditions associated with inflammation in otherwise healthy individuals [24].

### 2.5 Measurement of Lipids

Serum lipid levels comprising total cholesterol (TC), high density lipoprotein cholesterol (HDL-C) and low density lipoprotein cholesterol (LDL-C) were determined nine months after initial recruitment, on a Mindray BS120 machine as reported previously [21]. A randomly selected sub-population of the original cohort [8, 21] comprising the minimum sample size (N=152) was used in the current sub-study on lipids and inflammatory markers due to resource constraints.

### 2.6 Classification

Body mass index (BMI), an indicator of obesity, was computed using the formula: body weight/mass^2^ (kg/m^2^) and categorized according to World Health Organisation recommendations [8]. Dyslipidemia and hypertension were defined using United States of America’s National Cholesterol Education Program, Adult Treatment panell III guidelines [8]. Cytokine cut-off points were adopted from within range of existing studies for uniformity and comparability of results as follows: TNFα: 0.0-32.5 pg/ml [23, 25, 26], IL-10: 0.4-2.0 pg/ml [25, 26], levels of hsCRP were <1, 1 and>3 mg/dl corresponded to low, moderate and high cardiovascular disease risk [27].

### 2.7 Statistical Analysis

All statistical analyses were computed using STATA 13 (Stata Corp, College Station, Texas, USA) and Statistical Package for Social Science (SPSS) 21.0 for Windows. Demographic characteristics were summarised using mean and standard deviation (SD) for normally distributed data and median and interquartile range (IR) for non-Gaussian data. Comparison of means across groups was done using student’s t and analysis of variance (ANOVA) tests, depending on number of comparative groups. Comparison of medians was done using non-parametric k median tests for two groups and Kruskal-Wallis tests for more than two groups.

For comparison of more than two groups, Bonferroni post-hoc p-value was calculated according to number of comparative groups as 0.05/number of groups. Correlation between variables (inflammatory markers versus lipid fractions) was determined using bivariate regression analysis.

## 3 RESULTS AND ANALYSIS

The study population (N=152) consisted of both male (26%, n=39) and female (n=113) HIV infected patients, 82% (n=124) were ART-experienced and plasma samples were collected nine months after initial recruitment. Mean age of participants was 39 ± 9.3 years and males were generally older. Patients on ART had been on treatment for at least nine months and for an average of 3.7 years at the time of study. Median viral load indicated that most patients were virally suppressed though 9% (n=14) had elevated viral load (>1000 counts per ml). Mean CD4 count indicated that patients were generally immune competent with average CD4 counts >500 cells/mm^3^ at their lowest (nadir) level and at the time of sampling. However, approximately 32% (n= 49) were immune-compromised as indicated by CD4 count <500 cells/mm^3^.

Table **1** shows demographic, clinical and biochemical data for the population studied. Mean (SD) and median (IR) were within normal ranges for BMI, SBP, DBP, TC, HDL-C and TC/HDL-C ratio and pro-inflammatory cytokine TNF-α level. The median values for anti-inflammatory IL-10 and inflammatory protein marker hsCRP were elevated. Participants showed differences in age, BMI, TNFα and TNFα/IL10 ratio when compared according to sex (Table **2**). Males were generally older and had higher levels of TNFα and TNFα/IL10 whereas females had higher BMI. When participants were stratified by ART exposure (Table **3**), TC level was significantly higher (p=0.0005) in ART-experienced patients compared to ART-naive patients, HDL-C was significantly higher (p=0.0003) in ART-experienced patients and the TC/HDL-C ratio was not different between the two groups. Anti-inflammatory IL-10 levels were significantly higher in ART-experienced patients than in ART-naive patients, p=0.020, while pro-inflammatory TNF-α levels and TNF-α/IL10 ratios were significantly lower in ART-experienced patients compared to ART-naïve patients (p<0.0001, respectively).

Table **4** shows frequency of patients with elevated BMI, SBP, DBP, TC, HDL-C, TC/HDL-C ratio, TNF-α, TNFα/IL10 ratio, IL-10 and hsCRP in the whole group and in females only (males too few for sub-group analysis). Of importance from Tables **3** and **4** is that 7% of all patients were underweight (low BMI) while 37% of all patients and 42% of females and 43% of the ART-experienced were overweight (BMI>25). Proportion of patients with CD4 above 500 cells/mm^3^ had increased at the time of current study showing recovery in immune function for the patients. When patients were stratified by lipid levels, the group with normal HDL-C levels had significantly higher IL-10 levels than the group with low HDL-C levels (p=0.03). The most prevalent form of lipid dysfunction was depressed HDL-C (being over 60% for all patients and women only) (Tables **3** and **4**).

When inflammatory marker levels (TNF-α, IL-10, TNF-α/IL-10 ratio and hsCRP) were related to ART duration (Table **5**), stratified as: 0 years (ART-naives); 0-1 years, 1-5 years, 6-9 years and more than 10 years (10+ years), significant differences (p<0.05) were observed for TNF-α levels (p<0.0001) and TNF-α/IL-10 ratio levels (p<0.0001) dependent on the ART duration, even after Bonferroni post-hoc adjutment. Noteworthy, the number of subjects in each of the ART duration groups was low, especially for hsCRP and for the group of more than 10 years duration. Levels of inflammatory markers were also related to ART types (Table **6**). The ART naive group had the highest TNF-α value and the lowest IL-10 level compared to other types of ART. Interestingly, the group on PI-based second line regimen had the lowest TNF-α but the highest IL-10 value.

When inflammatory markers and lipids (TC, HDL-C, LDL-C, TC/HDL-C ratio) were tested for correlation, there was positive correlation only between IL-10 and TC, R^2^ =0.026, P=0.048 (Table **7**). No other correlations were found.

## 4 DISCUSSION

The current study is one of a few showing the plasma levels of selected inflammatory markers and traditional lipid levels in ART naive and ART experienced HIV infected patients in an African setting. This sub-study comprised 152 HIV patients who were part of a cohort of 215 patients reported earlier [8]. For the whole group, TNF-α levels were within reference range (0.1-32.5 pg/ml), while anti-inflammatory IL-10 levels were elevated (reference range; 0.4-2.0 pg/ml) which is consistent with findings in earlier studies from Western countries [28-30] and in two African studies [31, 32]. In the study from South Africa only 1.8% (n=7) of the participants had elevated TNFα levels, with medians similar across all HIV status strata in agreement with our findings (Tables **2**-**5**) [32]. In all studies elevated IL-10 levels could be associated with HIV independent of co-morbidities or persistent pathological level of T-cell activation in HIV infected patients [33].

Mean level of pro-inflammatory TNF-α was normal. According to literature, release of TNF-α, a major pro-inflammatory cytokine, is triggered by HIV infection in an attempt to eradicate viremia [11, 34, 35]. Incidentally, antiretroviral treatment will reduce the level of inflammation [36] which could be in accordance with our results as pro-inflammatory TNF-α levels were significantly higher in ART-naïve patients than in ART-experienced patients, p<0,0001. Literature reports that ART reduces the inflammation but that the inflammation persists even if the viremia is reduced to undetectable levels [28, 37-39]. Furthermore, we noticed that patients with the longest period of ART had the lowest TNF-α levels confirming that ART exposure could be associated with a reduction in pro-inflammatory marker levels (Table **6**).

Most patients in our Zimbabwean study were on first line ART regimens containing NNRTIs and NRTIs; only 7.24% were on protease inhibitor-based second line regimen. In accordance with our results earlier studies have shown that different ART combinations are associated with varying levels of inflammation (Table **7**). For example, Papakonstantinou *et al*. compared levels of inflammatory mediators for two groups of ART-naive patients placed on different first line regimens who were followed up for over 12 months [40]. According to their study, participants taking ART regimens that included tenofovir had lower levels of inflammatory mediators than those on ART regimens containing abacavir, suggesting a higher association with anti-inflammatory activity for ART regimens including tenofovir [40]. In the current study, patients on regimens containing tenofovir (comprising tenofovir, lamivudine and either nevirapine or efavirenz) had lower levels of inflammatory markers, when compared to the ART-naive group (Table **7**). Surprisingly, in the current study, participants on protease inhibitor-based second line regimens had the lowest levels of pro-inflammatory markers and the highest levels of anti-inflammatory markers further suggesting an association between some ART types and anti-inflammatory activity, in contrast to the SMART study [41], but in agreement with two other Western studies [42, 43].

The level of IL-10, an anti-inflammatory cytokine, was significantly elevated (p=0.020) in ART-experienced patients compared to ART-naive patients in this study, which is in contrast to a report from another Zimbabwean study [44]. The difference may be due to differences in time points at which these studies were carried out: Gori *et al.* [44] at baseline and our current study at follow-up, on sub-populations of the same cohort [8, 22]. Our study supports the hypothesis that patients on antiretroviral treatment have reduced levels of inflammatory markers, a possible mechanism for control of inflammation in ART positive patients [28, 36]. The Norwegian study reports a similar increase in IL-10, which is correlated to the progression in HIV infection and found that ART is associated with a decrease in IL-10 levels, but the levels remain elevated in the ART positive patients [28]. Even other studies have demonstrated that pro-inflammatory markers do not completely normalize in HIV infected ART positive individuals, suggesting that there is an on-going inflammation despite undetectable virus [28, 39]. In contrast to earlier studies [28, 45] we found that the TNF-α/IL-10 ratio was low in the ART positive as well as in the ART negative patients.

The plasma level of high sensitivity CRP was elevated in all the HIV infected patients in agreement with another South African study [31]. A substantial body of evidence associates this inflammatory marker with increased coronary heart disease risk [24, 44, 45]. There were, however, no association between hsCRP, TNF-α or IL-10 levels with most lipids (TC, LDL-C, HDL-C, TC/HDL-C ratio) in our current study. The only association found was between anti-inflammatory IL-10 and TC (r2=0.026, p=0.048).

We found that HDL-C levels were generally low in all the HIV positive patients, with lower levels of HDL-C in ART-naive patients compared to ART-experienced patients. Significantly lower HDL-C levels in ART-naive patients have previously been documented in other African [8, 46, 47] and Western studies [48, 49]. Higher TC levels were observed in ART-experienced patients compared to ART-naive patients (Table **4**), though average TC levels were within normal range. Gomo *et al*. studied impact of second-line antiretroviral regimens on lipid profiles in a Zimbabwean population [5]. In their study lipid levels were generally low after exposure to predominantly triple nucleoside first line ART regimen before switch to second line regimen [16, 49]. In our study the levels of TC and LDL were all within normal range, but were found to be slightly higher than the values found by Gomo *et al.* [5]. The difference could be due to the fact that in our study the participants had been on ART for a longer period and been treated over time by different combinations of ART. High TC, LDL-C, TC/HDL-C and low HDL-C are known to indicate increased cardiovascular disease risk [16]. We found that males had slightly higher LDL-C levels than females though the difference was not statistically significant (Table **3**). This was in contrast to the general observation that males are associated with more atherogenic lipid profile than females [16].

### 4.1 Limitations of the Study

The major limitation arises from the cross-sectional study design as no definite conclusions can be drawn from any links between variables. The patient groups are small, especially the ART negative groups and most participants were females. The different ART combinations described here are based on treatment at baseline. During the treatment time of ART there tended to be a shift of ART combinations for some of the patients either due to side effects of the medicine or due to progression of the HIV disease [21]. Use of different sample sizes for some assays due to resource constraints make comparisons difficult.

### 4.2 Strengths of the Study

This is one of the few African HIV studies to show association between inflammatory markers and lipid markers related to CHD and, furthermore, the influence of ART duration on the inflammatory and the lipid parameters. The patients were randomly selected and closely monitored for adherence and evidence of co-morbidities. Hence confounding by pre-existing opportunistic infections was reduced.

## CONCLUSION

The elevated levels of the two inflammatory markers IL-10 and hs CRP are suggestive of potential coronary heart disease risk in the HIV infected patients. Correlations between IL-10 and TC levels as well as differences between IL-10 levels in high and low HDL-C groups indicate a link between inflammatory markers and traditional markers of coronary heart disease risk. However, the use of inflammatory markers as independent coronary heart disease risk markers in HIV positive patients cannot be recommended until further studies and stronger evidences have been provided.

## Figures and Tables

**Table 1:** Demographics, clinical and biochemical data for all participants.

	**Variable**	**Mean**	**SD**	**Reference Ranges**
Demographics N=152	Age (years)	39	9.3	-
	BMI (kg/m^2^)	24.7	4.98	18.6-24.9
	SBP (mmHg)	122.8	17.8	<130
	DBP (mmHg)	79.6	15.7	<85
	Years on ART n=124 ART^+^ n=28 ART^-^	3.7 Naïve	3.4 Naïve	-
-	-	**Median**	**IR**	-
Clinical Data N=152	Viral load/copies/mL	37	20-58	0
	Nadir CD4 count (cells/mm^3^)	505.0	253.2	>500
	CD4 at time of study (cells/mm^3^)	693.3	303.1	>500
-	-	**Median**	**IR**	-
Biochemical Data (Lipids) N=152	LDL-C (mmol/L)	2.4	1.5-3.22	<2.59
	TC (mmol/L)	4.5	2.1-7.4	<5.17
	HDL-C (mmol/L)	1.1	0.9-1.4	>1.03 Males >1.29 Females
	TC/HDL-C ratio	3.9	3.1-4.5	<4.5
Biochemical Data (Inflammatory Markers) N=152	IL10 (pg/ml)	3.7	3.6-3.8	0.4–2.0 [23, 25, 26]
	TNFα (pg/ml)	12.3	10.5-16.4	0.0–32.5 [25, 26]
	TNFα/IL-10 ratio	3.5	-	-
	hsCRP (mg/dL) [N*=65]	3.8	1.7- 8.8	0-3 [24]

N, total number of study participants; N*, fewer participants; n, number of study participants per sub-group; SD, standard deviation; IR, interquartile range; BMI, body mass index; SBP, systolic blood pressure; DBP, diastolic blood pressure; TC, total cholesterol; HDL-C, high density lipoprotein cholesterol; LDL-C, low density lipoprotein cholesterol; IL-10, interleukin 10; TNF-α. tumour necrosis factor-alpha; hsCRP, highly sensitive C reactive protein

**Table 2:** Demographics, clinical and biochemical data stratified by sex.

**Variable**	**Mean (SD)**		**P-value**
**Demographics (N=152)**	**Male** **n =39**	**Female** **n =113**	-
Age (years)	42.3 (11.4)	37.7 (8.57)	0.019**
BMI (kg/m^2^)	21.6 (3.56)	25.3 (4.99)	0.0003**
SBP (mmHg)	122.9 (18.4)	122.7 (17.7)	0.955
DBP (mmHg)	79.5 (13.5)	79.6 (16.2)	0.966
**Lipids (N=152)**	**Mean (SD)**		-
LDL-C (mmol/L)	2.8 (0.98)	2.4 (1.52)	0.476
TC (mmol/L)	4.4 (1.1)	4.5 (1.1)	0.634
HDL-C (mmol/L)	1.2 (0.5)	1.2 (0.3)	0.541
TC/HDL-C ratio	4.1 (1.4)	3.9 (1.1)	0.282
**Inflammatory Markers**	**Median (IR)**		-
IL10 (pg/ml) N=152	3.5 (3.0-4.0)	3.7 (3.2- 5.2)	0.396
TNFα (pg/ml) N=152	15.5 (12.4-16.4)	11.7 (10.3-16.3)	0.003**
TNFα/IL-10 ratio N=152	4.3 (3.7- 4.7)	3.3 (2.1-4.2)	0.001**
hsCRP (mg/dL) N*=65	2.9 (1.7-13.1)	3.8 (1.4-8.8)	0.506

N, total number of study participants; N*, fewer participants; n, number of study participants per sub-group; SD, standard deviation; IR, interquartile range; TC, total cholesterol; HDL-C, high density lipoprotein cholesterol; LDL-C, low density lipoprotein cholesterol; IL-10, interleukin 10; TNF-α. tumour necrosis factor-alpha; hsCRP, highly sensitive C reactive protein; Starred P-values** designate significant P-value <0.05, where β= 0.2 and α=0.05 at 95% CI

**Table 3:** Lipid and inflammatory parameters stratified by ART experience.

**Variable**	**Mean (SD)**		**P-value**
N=152	ART naïve n=28	ART experienced n=124	-
**Lipids**	-	-	-
LDL-C (mmol/L)	2.3 (0.76)	2.5 (0.98)	0.315
TC (mmol/L)	3.8 (0.8)	4.6 (1.1)	0.0005**
HDL-C (mmol/L)	0.9 (0.3)	1.3 (0.4)	0.0003**
TC/HDL-C ratio	4.1 (1.3)	3.9 (1.2)	0.404
-	**Median (IR)**		-
**Inflammatory Markers**	-		-
IL-10 (pg/ml)	3.5 (3.1-3.3)	3.8 (3.2-4.9)	0.020**
TNF-α (pg/ml)	15.9 (14.9 -17.7)	11.6 (10.2-15.7)	<0.0001**
TNF-α/IL-10 ratio	4.5 (3.7-4.9)	3.3 (2.0-4.1)	<0.0001**
hs CRP (mg/dL) N*=65	2 (1-3.9)	3.8 (2.2-8.9)	0.417

N, total number of study participants; N*, fewer participants; n, number of study participants per sub-group; SD, standard deviation; IR, interquartile range; TC, total cholesterol; HDL-C, high density lipoprotein cholesterol; LDL-C, low density lipoprotein cholesterol; IL-10, interleukin 10; TNF-α. tumour necrosis factor-alpha; hsCRP, highly sensitive C reactive protein; Starred P-values** designate significant P-value <0.05, where β= 0.2 and α=0.05 at 95% CI

**Table 4:** Classification of patients by demographics, clinical and biochemical data for all patients and females only (males too few for subgroup analysis).

**Variable (ALL) N=152**	**Frequency**			**Reference Ranges**
	**Below normal**	**Elevated**	**Normal**	-
**Demographics (N=152)**	**n(%)**	**n(%)**	**n(%)**	-
BMI (kg/m^2^)	10 (7)	56 (37)	86 (56)	18.6-24.9
SBP (mmHg)	N/A	48 (32)	104 (68)	≥130
DBP (mmHg)l2	N/A	47 (31)	105 (69)	≥85
**ART History, N=152**	-	**Naïve**	**Experienced**	-
Frequency/n(%)	N/A	28 (18)	124 (82)	N/A
**Clinical data (N=152)**	**n(%)**	**n(%)**	**n(%)**	-
Nadir CD4 (cells/mm^3^)	146 (96)	N/A	6 (4)	>500
CD4 at time of study (cells/mm^3^)	49 (32)	N/A	103 (68)	>500
Viral load (counts per mL)	N/A	14 (9%)	138 (91)	<1000
**Lipids (N=152)**	**n(%)**	**n(%)**	**n(%)**	-
LDL-C (mmol/L)	N/A	48 (32)	104 (68)	>2.59
TC (mmol/L)	N/A	8 (6)	144 (94)	>5.17
HDL-C (mmol/L)	91 (60)	N/A	61 (40)	<1.03Males <1.29 Females
TC/HDL-C ratio	N/A	38 (25)	114 (75)	>4.5
**Inflammatory Markers**	**n(%)**	**n(%)**	**n(%)**	-
IL10 (pg/ml) N=152	N/A	60 (39)	92 (61)	0.4–2.0 [23, 25, 26]
TNFα (pg/ml) N=152	N/A	6 (4)	146 (96)	0.0–32.5 [25, 26]
hsCRP (mg/dL) N*=65	N/A	58 (38)	94 (62)	0-3
**Variable (FEMALES ONLY) N=113**	**Frequency**			**Reference Ranges**
-	**Below normal**	**Elevated**	**Normal**	-
**Demographics (N=113)**	**n(%)**	**n(%)**	**n(%)**	-
BMI (kg/m^2^)	3 (3)	49 (43)	61 (54)	18.6-24.9
SBP (mmHg)	N/A	34 (30)	79(70)	≥130
DBP (mmHg)	N/A	34 (30)	79 (70)	≥85
**ART History (N=113)**	-	**Naïve**	**Experienced**	-
Frequency/n(%)	N/A	19 (17)	94 (83)	-
**Clinical Data (N=113)**	**n(%)**	**n(%)**	**n(%)**	-
Nadir CD4 (cells/mm^3^)	90 (80)	N/A	23 (20)	>500
CD4 at time of study (cells/mm^3^)	23 (20)	N/A	90 (80)	>500
Viral load (counts per mL)	N/A	9 (8)	104 (92)	<1000
**Lipids (N=113)**	**n(%)**	**n(%)**	**n(%)**	-
LDL-C (mmol/L)	N/A	47 (42)	66 (58)	>2.59
TC (mmol/L)	N/A	7 (6)	106 (94)	>5.17
HDL-C (mmol/L)	72 (64)	N/A	41 (36)	<1.03Males <1.29 Females
TC/HDL-C ratio	N/A	24 (21)	89 (79)	>4.5
**Inflammatory Markers**	**n(%)**	**n(%)**	**n(%)**	-
IL10 (pg/ml) [N=113]	N/A	47(42)	66 (58)	0.4–2.0 [23, 25, 26]
TNFα (pg/ml) [N=113]	N/A	6 (5)	107 (95)	0.0–32.5 [25, 26]
hsCRP (mg/dL) [N*=43]	N/A	15 (35)	28 (65)	0-3

N, total number of study participants; N*, fewer participants; n, number of study participants per sub-group; TC, total cholesterol; HDL-C, high density lipoprotein cholesterol; LDL-C, low density lipoprotein cholesterol; IL-10, interleukin 10; TNF-α. tumour necrosis factor-alpha; hsCRP, highly sensitive C reactive protein

**Table 5:** Comparison of median levels of inflammatory markers by ART duration.

**ART duration**	**0 years**	**<1years**	**1-5years**	**6-9years**	**10^+^years**	**P-value
**Cytokines (N=152)**	Median (IR) n=27	Median (IR) n=29	Median (IR) n=58	Median (IR) n=33	Median (IR) n=5	-
TNF-α (pg/ml)	158.5 (149.1-175.1)	113.3 (107.1-167.4)	119.3 (97.3-160)	112.4 (101.8-152.3)	97.54 (95.4-105.6)	<0.0001
IL-10 (pg/ml)	34.94 (31.3-39.5)	37.6 (29.4-46.1)	37.6 (31.8-46.2)	43.2 (33.9-58.3)	35.54 (33.9-75.5)	0.143
TNF-α/ IL-10 ratio	4.52 (3.74-4.81)	3.36 (2.67- 3.99)	3.67 (1.58-4.20)	3.02 (1.67-3.49)	2.95 (1.29-2.97)	<0.0001
Inflammatory Marker (N*=65)	n=8	n=12	n=31	n=12	n=2	-
hs CRP (mg/L)	2.0 (1- 3.95)	2.9 (2.2-3.75)	3.8 (1.1- 7.7)	9.9 (4.1- 17.7)	5.1 (3.4-9.8)	0.106
**Sex (N=124)** n(%)	**Males** 30 (24)			**Females** 94 (76)		

N, total number of study participants; N*, fewer participants; n, number of participants per sub-group; IR, interquartile range; IL-10, interleukin 10; TNF-α. tumour necrosis factor-alpha; hsCRP, highly sensitive C reactive protein, **Significant Bonferroni post-hoc P =0.01, calculated for five comparative ART groups, where β= 0.2 and α=0.05 at 95% CI

**Table 6:** Comparison of median levels of inflammatory markers by ART type.

Marker	ART-naive	TDF/ NVP/ 3TC	TDF/ EFV/ 3TC	STV or ZDV +NVP/3TC	2^nd^ Line	P-value
-	n=27 (17.8%)	n=91 (59.9%)	n=17 (11.2%)	n=6 (3.9%)	n=11 (7.2%)	-
TNF-α (pg/ml)	159.1(149.9- 176.5)	117.1 (103.4- 154.7)	Median (IR) 111.7 (105.6- 172)	114.1(31.1- 147.2)	97.5 (47.1- 162.5)	0.029**
IL-10 (pg/ml)	34.9(30.6- 40)	36.6 (31.8- 47.5)	41.6(36.5- 59.5)	39.1(27.8-49.6)	43.4 (32.4-56.9)	0.001**
TNF-α/IL-10	4.53(3.70- 4.90)	3.38 (2.27- 4.18)	2.89(2.08-3.09)	3.81 (0.63- 4.54)	2.95(0.71- 3.54)	0.002**
-	n=8(5.26%)	n=42(27.63%)	n=7(4.61%)	n=3(1.97%)	n=5(3.29%)	-
hs CRP(mg/dl)	2(1- 3.95)	3.8 (1.4- 8.2)	3.4 (2.9-13.9)	6.5 (2.2-8.9)	14.3 (3.8-14.5)	0.481

N, number of participants per sub-group; IL-10, interleukin 10; TNF-α, tumour necrosis factor-alpha; hsCRP, highly sensitive C reactive protein, Starred P-values** designate significant P <0.01, calculated for five comparative ART groups, where β = 0.2 and α=0.05 at 95% CI

**Table 7:** Correlation analysis between inflammatory parameters and classical cardiovascular risk markers in HIV infected individuals; Correlation analysis was done using bivariate regression analysis.

Y Variables Inflammatory Marker	X Variables Lipids	R^2^	P-value
TNF-α (pg/ml) vs.	TC	0.0004	0.807
-	HDL-C	0.004	0.441
-	LDL-C	0.012	0.181
-	TC/HDL-C	0.0008	0.731
IL-10 (pg/ml) vs.	TC	0.026	0.048*
-	HDL-C	0.005	0.386
-	LDL-C	0.004	0.450
-	TC/HDL-C	0.0008	0.726
TNF-α/IL-10 vs.	TC	0.008	0.281
-	HDL-C	0.006	0.334
-	LDL-C	0.003	0.50
-	TC/HDL-C	0.0000	0.963
hsCRP (mg/dL) vs.	TC	0.022	0.243
-	HDL-C	0.006	0.529
-	LDL-C	0.002	0.733
-	TC/HDL-C	0.004	0.621

IL-10, interleukin 10; TNF-α, tumour necrosis factor-alpha; hsCRP, highly sensitive C reactive protein; TC, total cholesterol; HDL-C, high density lipoprotein cholesterol; LDL-C, low density lipoprotein cholesterol; IL-10, interleukin 10; TNF-α, tumour necrosis factor-alpha; hsCRP, highly sensitive C reactive protein; BMI, body mass index; R^2^ = coefficient of determination, Starred P-value* designates significant P <0.05;where β= 0.2 and α=0.05 at 95% CI

## LIST OF ABBREVIATIONS

AIDS: = Acquired Immune Deficiency Syndrome
ART: = Anti-Retroviral Therapy
BMI: = Body Mass Index
CHD: = Coronary Heart Disease
CVD: = Cardiovascular Disease
CD4: = Cluster of Differentiation 4
CD8: = Cluster of Differentiation 8
DBP: = Diastolic Blood Pressure
EFV: = Efavirenz
ELISA: = Enzyme-Linked Immuno-sorbent Assay
HDL-C: = High Density Lipoprotein Cholesterol
HIV: = Human Immunodeficiency Virus
hsCRP: = highly sensitivity C-reactive protein
IL: = Interleukin
IR: = Interquartile Range
JREC: = Joint Research Ethics Committee
LDL-C: = Low Density Lipoprotein Cholesterol
LPV/r: = Lopinavir/ritonavir
mg/dl: = Milligrams per decilitre
MI: = Myocardial Infarction
ml: = millilitre
mmol/L: = millimoles per litre
MRCZ: = Medical Research Council of Zimbabwe
NCEP: = National Cholesterol Education Program
Nef: = Negative Factor
NF-k B: = Nuclear Factor kappa B
NK: = Natural Killer
NNRTI: = Non-Nucleoside Reverse Transcriptase Inhibitor
NRTI: = Nucleoside Reverse Transcriptase Inhibitor
NVP: = Nevirapine
PI: = Protease Inhibitor
REK: = Research Ethics Committee
SBP: = Systolic Blood Pressure
SD: = Standard Deviation
sTNF-R1: = Soluble Tumour Necrosis Factor Receptor 1
STV(d4T): = Stavudine
tat: = Trans-Activator of Transcription
TC: = Total Cholesterol
TDF: = Tenofovir
TNF-α: = Tumour Necrosis Factor-alpha
VLDL-C: = Very Low Density Lipoprotein Cholesterol
ZDV: = Zidovudine
3TC: = Lamivudine
